# Infantile esotropia could be oligogenic and allelic with Duane retraction syndrome

**Published:** 2011-07-20

**Authors:** Arif O. Khan, Jameela Shinwari, Latifa Al Sharif, Dania Khalil, Saeed Al-Gehedan, Nada A. Al Tassan

**Affiliations:** 1 Division of Pediatric Ophthalmology, King Khaled Eye Specialist Hospital, Riyadh, Saudi Arabia; 2 Division of Anterior Segment, King Khaled Eye Specialist Hospital, Riyadh, Saudi Arabia; 3 Department of Genetics, King Faisal Specialist Hospital & Research Center, Riyadh, Saudi Arabia

## Abstract

**Purpose:**

To describe phenotyping and linkage analysis results for available members from a consanguineous nuclear family with hereditary congenital strabismus.

**Methods:**

Both parents and all 12 children underwent clinical examination. Available affected and several unaffected family members had venous blood sampling for DNA extraction and 10K single nucleotide polymorphism (SNP) genotyping (Affymetrix Gene Chip® Human). Multipoint logarithm of the odds (LOD) score calculations were performed assuming an autosomal recessive mode of inheritance with 100% penetrance and disease allele frequency of 0.01%.

**Results:**

Three children had non-syndromic large-angle infantile esotropia without significant hyperopia. A fourth child had left esotropic Duane retraction syndrome. A fifth child who had esotropia in the setting of prematurity and childhood poliomyelitis was excluded from the analysis. A sixth child had keratoconus and was excluded. Both parents and the remaining 6 children had no significant orthoptic or ophthalmic findings. Using linkage analysis including the 4 esotropic children, disease loci were mapped to regions on chromosomes 3p26.3–26.2 and 6q24.2–25.1 using multipoint linkage analysis with LOD scores of 3.18 and 3.25 respectively. Linkage to these regions persisted when the esotropic Duane retraction syndrome patient was excluded from the linkage analysis (LOD scores of 2.00 and 2.32, respectively).

**Conclusions:**

Non-syndromic infantile esotropia could be related to susceptibility loci on chromosomal regions 3p26.3–26.2 and 6q24.2–25.1 and may share alleles that underlie Duane retraction syndrome.

## Introduction

Strabismus (ocular misalignment) affects up to 4% of the general population [1]. Comitant strabismus is misalignment that does not significantly change during different positions of gaze; incomitant strabismus is misalignment that does change depending upon gaze direction. Although many cases of non-syndromic early childhood strabismus seem sporadic, recessive and dominant inheritance patterns can be inferred from familial cases [2,3]. In addition to genetic predisposition, both ocular and non-ocular factors can also cause strabismus [2].

Esotropia is the most common form in Western populations [1,2] and has several subtypes. Refractive accomodative esotropia is secondary to inappropriate convergence during accomodative effort in an uncorrected hyperope and is often familial. Infantile esotropia, nonsyndromic large-angle deviation noted within a few months of birth, is usually not associated with significant refractive error and is typically sporadic but can be familial [2,3]. Esotropic Duane retraction syndrome is a congenital cranial dysinnervation disorder in which the lateral rectus has subnormal innervation from the sixth cranial nerve and variable inappropriate innervation from the third cranial nerve; it is the most common form of congenital incomitant strabismus and is typically sporadic but can be familial [1,4]. One form of familial Duane syndrome can be caused by a heterozygous mutation in chimerin 1 (*CHN1*) [5].

Most advances in the genetics of ocular motility have come from studies of families with rarer forms of congenital incomitant strabismus, i.e., congenital cranial dysinnervation disorders such as congenital fibrosis of the extraocular muscles, horizontal gaze palsy with progressive scoliosis, and familial Duane syndrome [5,6]. Family studies have led to identification of genes such as kinesin family member 21A (*KIF21A*; dominant congenital fibrosis of the extraocular muscles), paired-like homeobox 2a (*PHOX2A*; recessive congenital fibrosis of the extraocular muscles), roundabout homolog 3 (*ROBO3*; recessive horizontal gaze palsy with progressive scoliosis), and *CHN1* (dominant Duane retraction syndrome) [5,6]. Affected consanguineous families with a relatively large number of affected children more commonly have recessive cause for familial ocular disease and have facilitated the uncovering of genes associated with recessive congenital cranial dysinnervation disorders.

In contrast, genetic mechanisms underlying common forms of comitant esotropia are less well described. One reason is genetic heterogeneity for early childhood esotropia [1-3,7]. Different genotypes can underlie the same strabismus subtype, those cases that are genetic may be oligogenic rather than monogenic, and environmental factors such as prematurity can also play a strong independent role. Another reason is that familial early childhood esotropia is rarely encountered in the types of families most amenable to genetic analysis – large consanguineous ones with several affected children. In addition, prior genetic studies of comitant strabismus do not always carefully phenotype family members and often group separate subtypes of strabismus as a single phenotype [8-11]. For example, accommodative esotropia is often grouped with infantile esotropia although the former may be secondary to an uncorrected high hyperopic refraction while the later is a primary infantile defect in ocular alignment.

In the current study we report results of careful ophthalmic phenotyping and linkage analysis for available members of large consanguineous nuclear family in which several siblings had early childhood esotropia.

## Methods

This study was approved by our our institutional review boards (those of the King Khaled Eye Specialist Hospital and King Faisal Specialist Hospital & Research Center, Riyadh, Saudi Arabia) and adhered to the tenets of the Declaration of Helsinki. Full informed consent was obtained from the family after explanation of the nature of the study.

### Clinical

The 14-member consanguineous family was identified from the pediatric ophthalmology practice of one of the authors (A.O.K.) and was invited to participate in the study. Each family member underwent complete ophthalmic examination with attention to ocular motility (e.g., asymptomatic microtropia) both before and after pharmacologic cycloplegia (cyclopentolate 1%) by an ophthalmologist with strabismus experience (A.O.K.).

### Genetic

Venous blood samples (3–5 ml) for DNA analysis were collected from all affected individuals, the unaffected parents, and available unaffected individuals. Extraction of DNA was by Genetra systems (QIAGEN, Valencia, CA) according to manufacturer conditions. Genotyping of single nucleotide polymorphisms (SNPs) was performed as detailed by Affymetrix (Santa Clara, CA) on their GeneChip^®^ Human Mapping 10K Array Xba 142 2.0. SNP genotypes were called using Affymetrix GCOS 1.4 software with an overall SNP call rate of 95%–99%. Multipoint logarithm of the odds (LOD) score calculations were performed with the Allegro module of the Easy Linkage software package [12] assuming an autosomal recessive mode of inheritance with 100% penetrance and disease allele frequency of 0.01%.

## Results

### Clinical

**Figure 1:**
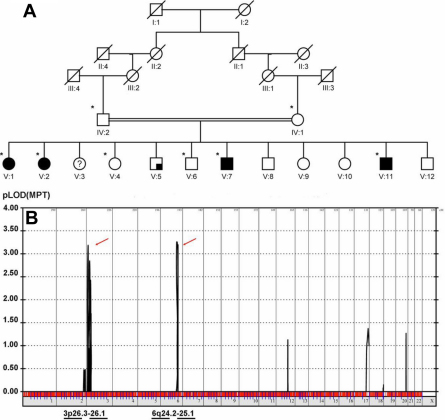
Initial linkage analysis. **A**: The family pedigree. Analyzed patients are indicated with asterisks. Full coloration indicates infantile esotropia or Duane retraction syndrome; quarter coloration indicates keratoconus. **B**: Multipoint linkage analysis (the unaffected parents, all 4 esotropic siblings, and 2 unaffected siblings) revealed maximum logarithm of odds (LOD) scores on chromosomes 3p26.3–26.1 (LOD score 3.18) and 6q24.2–25.1 (LOD score 3.25).

Five siblings had ocular complaints. One asymptomatic family member had significant ophthalmic findings (V:7) while the other asymptomatic family members did not; those with ophthalmic findings are labeled in Figure 1. Individuals with significant ocular findings are summarized below; those considered to be affected are in bold italics.

#### V:1

This 27-year-old female had large-angle esotropia noted within the first few months of life and an unremarkable birth history. Examinations by pediatric ophthalmologists and orthoptists during the first few years of life confirmed the diagnosis of infantile esotropia (45 prism diopters) with full ductions, a strong fixation preference for the right eye, and no significant hyperopia. During early childhood she underwent left medial rectus recession (5 mm) and left lateral rectus resection (8 mm) and developed left exotropia in the post-operative period. Examination at 27 years of age was remarkable for amblyopia in the left eye (20/20 OD, count fingers OS), a left exotropia of 35 prism diopters, and slight limitation of adduction OS. Cycloplegic refraction of both eyes was approximately plano and there was no structural ocular abnormality.

#### V:2

This 26-year-old female had large-angle esotropia noted within the first few months of life and an unremarkable birth history. Examinations by pediatric ophthalmologists and orthoptists during the first few years of life confirmed the diagnosis of infantile esotropia (45 prism diopters) with full ductions, alternating fixation, and no significant hyperopia. During early childhood she underwent right medial rectus recession (5 mm) and right lateral rectus resection (8 mm). Examination at 26 years of age was significant for 20/25 vision in both eyes, esotropia of less than 10 prism diopters with mild dissociated vertical deviation in both eyes, and a cycloplegic refraction of plano OD and −1.00 OS.

#### V:3

This 25-year-old female was noted to have esotropia since early childhood. She was born 7 months premature and developed poliomyelitis in early childhood. Because of confounding medical issues, she was excluded from the analysis.

#### V:5

This 21-year-old male had no history of strabismus but complained of decreasing vision over the last several years. Birth history was unremarkable. Best-corrected visual acuity was 20/40 OD, 20/30 OS. Ophthalmic examination was significant for inferior paracentral corneal ectasia and a warped retinoscopy reflex (high myopia and astigmatism). This individual was diagnosed with keratoconus and excluded from the analysis.

#### V:7

This 16-year-old male was asymptomatic and had an unremarkable birth history. Visual acuity was 20/30 OD, 20/100 OS. Ophthalmic examination was significant in the left eye for mild limited abduction and moderate globe retraction during adduction, i.e., esotropic Duane retraction syndrome. Cycloplegic refraction was +4.00 OD and +5.00 in the left eye. There were no structural ocular abnormalities.

#### V:11

This 5-year-old boy had large-angle esotropia noted since birth and had an unremarkable birth history. Examination was significant for 20/30 vision in the right eye and count-fingers vision in the left eye, esotropia of 80 prism diopters, full ductions, a cycloplegic refraction of +1.00 OU, and no structural ocular defects.

### Genetic

Multipoint linkage analysis (the unaffected parents, all 4 esotropic siblings, and 2 unaffected siblings) identified multiple disease loci on chromosomes 3p26.3–26.2 (Ensembl cytogenetic band, rs1403635 and rs722368) with a maximum LOD score of 3.18 and 6q24.2–25.1 (Ensembl cytogenetic band, rs199242 and rs1977656) with a maximum LOD score of 3.25 (Figure 1). The region on chromosome 3 is of 2.46 MB and comprises 12 genes and the region on chromosome 6 is 6.9 MB in size with 62 genes.

**Figure 2:**
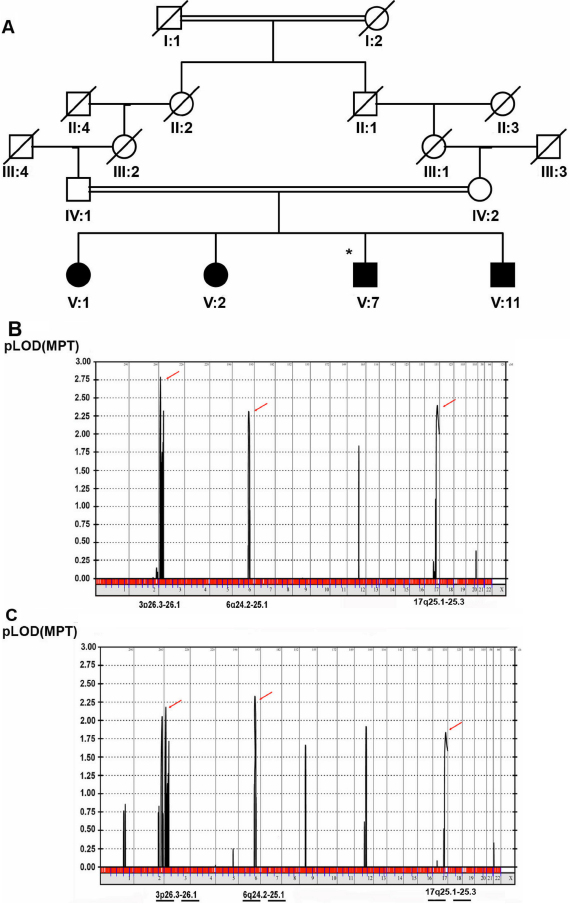
Subsequent linkage analyses. **A**: Affected children from the family. Full coloration indicates affected individuals; asterisk indicated esotropic Duane retraction syndrome (other affected individuals had infantile esotropia). **B**: Repeated linkage analysis with the same parameters but excluding the 2 unaffected siblings (i.e., using the parents and all 4 esotropic children) confirmed the linkage to the same regions on chromosomes 3 and 6 with LOD scores of 2.78 and 2.31. An additional candidate region of 5.5 MB in size on chromosome 17q25.1–25.3 with a LOD score of 2.39 was detected. **C**: Analysis without the esotropic Duane retraction syndrome patient (i.e., for the 3 infantile esotropes and the parents only) confirmed linkage to the same regions on chromosome 3 and 6 with LOD scores of 2.00 and 2.32 while the LOD score on chromosome 17 decreased to 1.83.

We repeated the linkage analysis with the same parameters but excluding the 2 unaffected siblings (i.e., using the parents and all 4 esotropic children). The result confirmed the linkage to the same regions on chromosomes 3 and 6 with LOD scores of 2.78 and 2.31; in addition, an additional candidate region of 5.5 MB in size on chromosome 17q25.1–25.3 with a LOD score of 2.39 was detected (Figure 2). When the same analysis was repeated without the esotropic Duane retraction syndrome patient (i.e., for the 3 infantile esotropes and the parents only), results confirmed linkage to the same regions on chromosome 3 and 6 with LOD scores of 2.00 and 2.32 while the LOD score on chromosome 17 decreased to 1.83 (Figure 2). To test for a potential locus for infantile esotropia that is not allelic to Duane retraction syndrome, analysis was repeated with the Duane retraction syndrome patient scored as unaffected. A locus of 4.9MB on chromosome 2q36.3–37.3 (Ensembl cytogenetic band, rs475525 and rs1822180) with a LOD score of 2.4 was detected.

## Discussion

Three siblings in this large consanguineous family had infantile esotropia and a fourth had Duane retraction syndrome. The linkage analysis results suggest oligogenic inheritance for this family’s infantile esotropia and that esotropic Duane retraction syndrome could be allelic to infantile esotropia.

To the best of our knowledge there are only 2 prior genetic studies that examined the specific strabismus phenotype of infantile esotropia: one which showed a 94.1% concordance for infantile esotropia in monozygotic twins [13], and a second that suggested a codominant model in many cases [3]. Other genetic studies have described strabismus susceptibility loci but often without differentiating among different strabismus subtypes. Both recessive and dominant linkage of childhood esotropia to chromosome 7p22.1 have been reported in one family each, but without differentiation of accommodative esotropia from infantile esotropia [8,9]. Additional susceptibility loci for comitant strabismus such as chromosomes 4q28.3 and 7q31.2 and chromosomes 6q26, 12q24.32, and 19q13.11 have been reported, but again various subtypes of strabismus were grouped as a single phenotype [10,11]. Our results suggest chromosomes 3p26.3–26.2 and 6q24.2–25.1 could contain susceptibility loci for familial infantile esotropia in a consanguineous nuclear family.

Like other forms of early childhood esotropia, Duane retraction syndrome is genetically heterogeneous and sometimes caused by environmental cause [1,14]. Approximately 70% of cases do not have other recognized congenital abnormality, and up to 20% of cases typically have a family history of strabismus [1,14]. Examples of Duane retraction syndrome with systemic findings and a known genetic basis include dominant mutation in Sal-like 4 (*SALL4*; with radial ray and/or renal malformation), dominant mutation in Sal-like 1 (*SALL1*; with renal, anal, and/or auricular malformation), or recessive mutation in homeobox A1 (*HOXA1*; with inner ear and cerebrovascular malformation and autism) [14]. The Duane retraction syndrome 1 (DURS1) locus has been localized to chromosome 8q13 based on cytogenic abnormalities observed in several patients with Duane retraction syndrome as a part of presumed contiguous gene syndrome [1,14] but no specific gene has been identified. The DURS2 locus was localized to chromosome 2q31 by linkage analysis of a large dominant non-syndromic pedigree and is now known to be *CHN1* [5]. However, most patients with Duane retraction syndrome do not have mutation in *CHN1* [15,16]. Two previous studies [17,18] hypothesized that isolated Duane retraction syndrome may in some families be allelic to infantile esotropia. Our linkage analysis results support this hypothesis for the current family.

In summary, for this consanguineous family we suggest oligogenic susceptibility loci for infantile esotropia and that Duane retraction syndrome could be allelic to infantile esotropia. Careful phenotyping and analyses in similar families are needed to further understanding of the genetics underlying common forms of strabismus.
